# In Vitro Assessment and Comparison of a Novel Electromagnetic Tracking System for Stereotactic DBS Surgery

**DOI:** 10.1007/s10439-025-03728-9

**Published:** 2025-04-10

**Authors:** Céline Vergne, Morgan Madec, Raphael Guzman, Joris Pascal, Ethan Taub, Frédéric Bourgeois, Simone Hemm

**Affiliations:** 1 School of Life Sciences, Institute for Medical Engineering and Medical Informatics, University of Applied Sciences and Arts Northwestern Switzerland, Muttenz, Switzerland; 2 ICube Laboratory, University of Strasbourg–CNRS, Strasbourg, France; 3 Department of Biomedical Engineering, Faculty of Medicine, University Basel, Allschwil, Switzerland; 4 Department of Neurosurgery, University Hospital Basel, Basel, Switzerland

**Keywords:** Electromagnetic tracking, Deep brain stimulation, Interventional guidance, System assessment, Stereotactic neurosurgery

## Abstract

Real-time guidance for the implantation of deep-brain-stimulation (DBS) electrodes in the context of stereotactic neurosurgery is essential but currently unavailable. Electromagnetic tracking (EMT) systems offer high-accuracy localization of tools in restricted volumes but face compatibility issues with stereotactic procedures due to electromagnetic distortions. This paper aims to evaluate and compare the localization performance (position and orientation) of a novel EMT system, the ManaDBS, specifically designed for stereotactic surgical environments, against the NDI Aurora, a commercially available EMT system. Two studies were conducted to assess the suitability of each EMT system for stereotactic DBS surgery. The first study evaluated performance accuracy within the measurement volume in the presence of two different stereotactic systems (Frame G and Vantage system, Elekta). The second study simulated a DBS surgical theater, performing implantation procedures with each EMT system and evaluating the position accuracy of the EMT sensor. The localization errors of Aurora (0.66 mm and 0.89°) were lower to those of ManaDBS (1.57 mm and 1.01°). However, in the presence of a stereotactic system, Aurora exhibited notable degradation (2.34 mm and 1.03°), whereas ManaDBS remained unaffected. This pattern persisted during simulated implantation in a DBS surgical environment, where nonlinear trajectories with significant error fluctuations along the implantation path were observed with Aurora system. The significant electromagnetic-field distortions render the Aurora system incompatible for stereotactic DBS surgery. However, the ManaDBS system exhibited no impact from these distortions, suggesting its potential suitability for DBS surgery and other potential applications in stereotactic neurosurgery.

## Introduction

DBS surgery involves implanting electrodes into deep brain structures to alleviate motor disorders such as Parkinson’s disease or essential tremor. Typically performed with a stereotactic system under local anesthesia, the procedure is complemented by microelectrode recording (MER) of the neuronal activity and/or through stimulation tests to verify the target location [1]. Intraoperative validation of the electrode implantation position can be achieved using 2D X-rays or 3D computed tomography (CT). However, challenges remain, such as the angular localization of new directional DBS electrodes or real-time navigation during implantation. A promising technique for addressing these challenges is electromagnetic tracking (EMT) [2, 3], which has been explored as a complementary or alternative imaging method to X-rays. EMT systems provide regular feedback on the position and orientation of DBS electrodes, offering advantages like reduced radiation exposure and precise 3D localization relative to brain structures. A comprehensive review by Frank in 2014 [4] presented an overview of the commercial market and typical EMT technology used. The state-of-the-art EM systems approved for neurosurgery include the AxiEM (Medtronic Inc., Minneapolis, USA) and the Kick EM (Brainlab, Munich, Germany). These systems are integrated into surgical platforms that also incorporate optical tracking and image-guided software, such as Medtronic’s StealthStation. Table 1 summarizes the known characteristics of the two systems. In the context for DBS electrode, a study by Burchiel [5] showed comparable performance between EMT and CT guidance. However, due to compatibility issues, frameless stereotactic surgery was performed instead of the standard stereotactic procedure.

**Table 1:** Description of the two EMT systems available on the market for neurosurgery: Medtronic AxiEM and the Brainlab Kick EM [22, 23]

EMT System	Medtronic AxiEM	Brainlab Kick EM (Model: NDI Aurora V3)
*Emitter type*	Flat and Side-mount	Side-mount
*FG dimension* *(approximate)*	Side-mount FG: 13.5 x 13.5 x 8.5 cm Flat FG: 50 x 35 x 3.5 cm	20 x 20 x 7 cm
*Magnetic field*	FG emits low intensity and varying electromagnetic fields which induce small currents in the sensors embedded in the instrument.	
*Volume of tracking*	Side-mount FG: 46 x 46 x 31 cm Flat FG: 40 x 40 x 37.5 cm	50 x 50 x 40 cm
*Sensors*	Micro-coils	
*Datasheet* *warnings*	Metallic and conductive objects such as the surgical table. Recommended distance from the metal of 5 cm for the flat emitter and 25 cm for the side-mount emitter.	System accuracy may be affected by the setup (monitor, base station...). Some metal objects and radio frequency communication equipment may cause interference.

Despite its potential, EMT technology has limitations that affect its clinical application. One inherent limitation is the minimum practical sizes of the sensors and field generators necessary for the optimal functioning of EMT systems. Another significant constraint arises from the technology’s sensitivity to electromagnetic (EM) distortions caused by the proximity of medical diagnostic devices like CT or MRI scanners [6], as well as ferromagnetic objects [7]. These issues accentuate the need for ongoing advancements to enhance EMT’s robustness and adaptability in clinical settings*.* Commercial EMT systems comprise a field generator (FG) that produces an alternating magnetic field and specialized magnetic sensors known as micro-coils. However, operating with alternating magnetic fields at frequencies in the hundreds of kilohertz introduces additional EM distortions. Beyond the primary sources of distortions, including ferromagnetic materials and electronic devices, conductive distortions must also be considered. These distortions arise from Eddy currents induced by alternating magnetic fields in conductive materials, which generate secondary EM fields that interfere with the primary magnetic field. This secondary EM field is present only during FG activation and operates at the same frequency, making it particularly challenging to compensate for. The magnitude of conductive distortions depends on many factors such as the size, composition, proximity, and shape of the distortion sources. Nixon et al. [8] proposed an initial theoretical model that correlates tracking errors with distortion sources. This model is based on experimental observations and is therefore tailored to the specific setup used. Given the unique nature of each surgical environment, translating performance assessments and compensation strategies [9, 10] across different setups remains challenging.

Concurrently with these advancements, there is a growing interest in EMT systems based on quasi-static magnetic fields. Over the past decade, progress in miniaturized integrated magnetic sensors has enabled the design of functionalized catheters with millimetric dimensions, offering enhanced mechanical robustness in contrast to micro-coils. The primary benefit of an EMT technique based on integrated magnetic sensors and quasi-static magnetic fields lies in its inherent resistance to conductive distortions and ability to maintain a high level of precision [11, 12]. Nevertheless, this EMT technology suffers from another limitation: its reduced update rate ranging from 1 to 10 Hz, which defines the number of localization outputs provided per second. This limited update rate restricts this EMT technology’s suitability to certain surgical procedures such as DBS surgery, as it involves slowly inserting electrodes along tens of centimeters into the patient’s brain. Knowing that many clinical centers heavily rely on stereotactic systems, the introduction of a compatible EMT system could enhance safety and reduce surgery duration. To this end, we developed a quasi-static EMT system, named ManaDBS, for intraoperative localization of DBS electrodes [13, 14].

This paper provides a comprehensive evaluation of the performance and a comparative analysis between the NDI Aurora V2 system and the ManaDBS system. The two studies focuses on the integration of each EMT system within a DBS surgical environment including a stereotactic system. The commercial system from NDI is a well-known electromagnetic tracking system. The V3 version of the NDI Aurora system is notably marketed by Brainlab under the product name Kick EM, as indicated in the FDA’s 510(k) premarket notification [15]. While the system used in this paper is an earlier version, the underlying technological principles remain the same. Through two distinct studies, we aimed to provide valuable insights on the applicability of EMT systems in the context of stereotactic DBS surgery.

## Materials & Methods

### Electromagnetic Tracking Systems

**Fig. 1:**
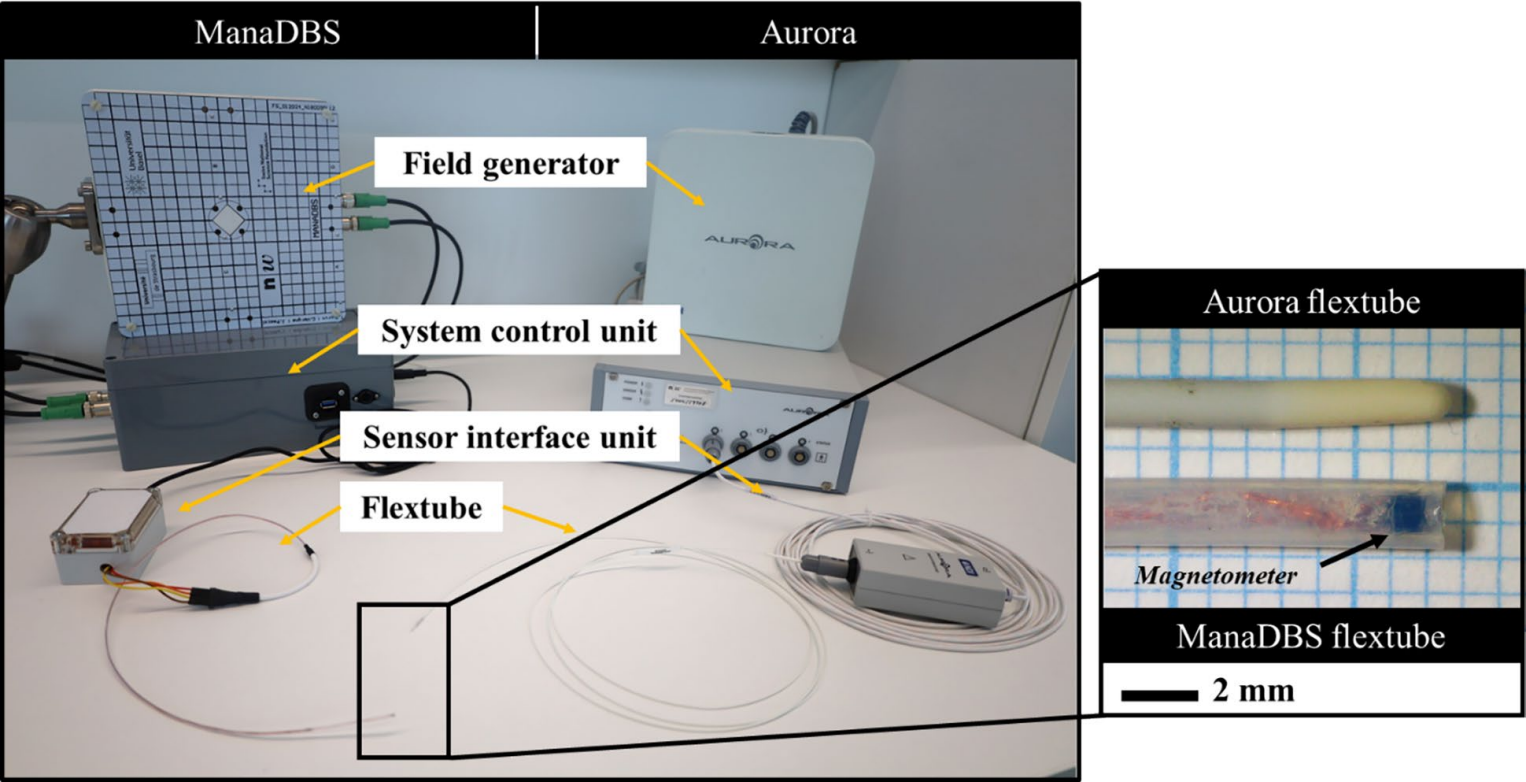
The two navigation systems: Aurora (right) and our developed system ManaDBS (left), both with a field generator of 20 × 20 × 7 cm and the associated flextubes with diameters of 1.3 mm and 1.8 mm, respectively

**NDI Aurora** The commercial EMT system considered in this paper is the Aurora V2 from Northern Digital Incorporated (NDI) (Waterloo, Canada), depicted in Figure 1. The FG, with dimensions of 20 × 20 × 7 cm, generates an alternating magnetic field within a tracking volume of 50 × 50 × 50 cm^3^. NDI reports localization errors of 0.5 mm and 0.3° at the center of the tracking volume with an update rate of 40 Hz. A flextube of 1.3 mm diameter, containing a standard six degrees-of-freedom (DOF) sensor (610060 from NDI) was used as the tracking tool in the two evaluative studies. All tracking data (positions and orientations) were recorded and managed via the NDI ToolBox software.

**ManaDBS** The technological principle of the ManaDBS system relies on quasi-dc magnetic field measured by monolithic magnetic sensors. The FG consists of four planar coils which sequentially generate static magnetic field with a maximum strength of 160 µT. To ensure consistency with the Aurora system and avoid introducing additional errors in the comparative study, the dimensions of the FG were made identical to those of the Aurora, allowing the two FGs to be swapped in the experimental setup. The activation sequence of the coils follows five steps: the first four steps involve activating each coil individually, and the fifth step involves offset cancelation. During offset cancelation, the environmental magnetic field, including the Earth’s magnetic field, is measured with all coils turned off. This environmental field data is subtracted from the first four measurements to eliminate any static perturbations. The outcome of the five-step sequence is a matrix of 15 magnetic field components, reduced to 12 magnetic components after offset cancelation. The FG is designed to uniquely encode each point in the measurement volume (MV) with a magnetic field amplitude vector. This unique encoding is based on the principle of multilateration, where an object’s position is determined by measuring signals from multiple transmitters. In the case of electromagnetic localization, multilateration uses the magnetic fields generated by the FG, which vary with the distance between the FG and the magnetic sensor. The update rate of ManaDBS is 0.3 Hz. To ensure high tracking accuracy, precise knowledge of the magnetic fields is required. Static magnetic field simulations were conducted using the Radia magnetostatic library in Python 3.8 [16]. Additionally, the current supply to the coils is regulated to ensure fluctuations remain within 1% at a supply current of 5 A. The detailed principle of the ManaDBS system and its characterization were presented in a previous publication [14], which reported localization errors of 1.72 mm and 0.89° within a tracking volume of 15 × 15 × 30 cm^3^. For this study, a customized flextube with a diameter of 1.8 mm was developed. The flextube contains an anisotropic magnetoresistive three-axis sensor (MMC5603 NJ, Memsic Semiconductor Co.) placed at its tip (Figure 1).

**Measurement volume** The tracking volumes of the EMT systems are larger than the defined measurement volume for the studies. Following the requirements of DBS surgery, the measurement volume was defined to be equivalent to the volume of the head, i.e., 15 × 15 × 15 cm^3^. The offset distance, defined as the distance between the FG and the closest point from the measurement volume, was set at 18 cm.

## Study 1: Tracking Performance in the Presence of a Stereotactic System

Study 1 evaluated the performance of both EMT systems within the measurement volume using a high-precision setup to accurately replicate experimental conditions. Three distinct settings were examined. Initially, we established a baseline environment free of any EM perturbations. Subsequently, we conducted two additional experiments introducing two different stereotactic systems: the Leksell Stereotactic G System (Elekta AB, Stockholm, Sweden), which has been a reference for functional neurosurgery and particularly for DBS surgery for over two decades, and the recently released Leksell Vantage Stereotactic System (Elekta AB). These three settings are referred to as the baseline, Gframe, and Vantage setups, respectively.

**Fig. 2:**
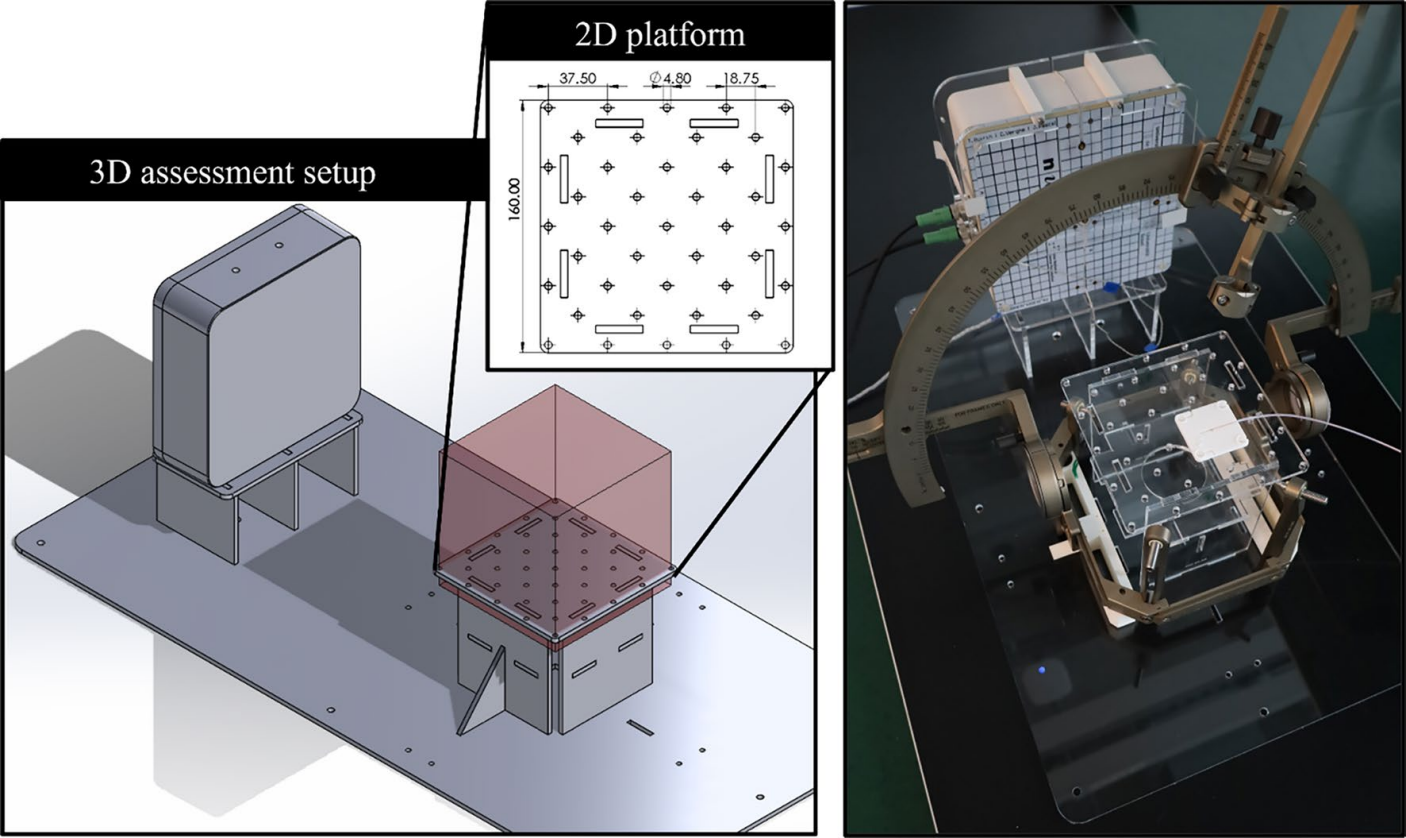
On the left, a rendering of the 3D assessment setup, constructed with 5 mm-thick PMMA. The measurement volume (15 × 15 × 15 cm^3^) is represented in red. On the right, a picture of the Gframe setup, including the Stereotactic G system (frame and arc) and the ManaDBS system

**3D assessment setup** Setups for assessing the performance of EMT systems are commonly made from Lego [17] or Polymethyl methacrylate (PMMA) arrangements [18, 19] to avoid additional EM distortions. A custom-made fixed-size 3D system was built using 5 mm PMMA plates (Figure 2). Four 2D platforms of varying heights were sequentially positioned at 18 cm from the FG. Each platform included 25 positions. A 3D-printed piece holding the flextube was manually placed at each position. A total of 100 positions were acquired, covering a volume of 11.25 × 11.25 × 11.25 cm^3^, as depicted in Figure 2. At each position, 25 measurements were acquired for each flextube. The stereotactic systems (frame and arc) were positioned around the platforms and secured using 3D-printed holders.

**Setup calibration** To validate the placement of the flextube within the assessment setup, we established reference positions using the NDI Optotrak Certus optical tracking system (Northern Digital Inc., Waterloo, Canada). This calibration step was essential to correct any errors introduced during the manufacturing of the 3D assessment setup. The NDI Optotrak Certus system provides a reported localization accuracy of 0.1 mm. Four LED markers were fixed on the FG, while four embedded markers were integrated into the flextube holder. These optical markers were employed to capture the 100 positions within the 3D assessment setup, which were then transformed into the coordinate system of the FG. Subsequently, the reference positions were aligned with the measured positions of the baseline setup using the iterative closest point (ICP) algorithm simpleICP [20]. The ICP algorithm accounted for any misalignment or errors arising from the placement of LED markers on the FG and flextube holder. Once the ICP registration was completed, the corrected reference positions were saved and used as the ground truth for evaluating localization performance.

**Data analysis** The measured positions and orientations were averaged over 25 measurements per location. Position errors were defined as the Euclidian distance between the measured position and the ground-truth position. The orientation error was also defined as the Euclidian distance (in the angular space) between the measured orientation and the true orientation. The latter was defined to be the average orientation measured over 100 positions in the baseline setup. The jitter for both position and orientation, was calculated as the standard deviation of all 25 measurements per location. Statistical analysis was performed using Python 3.9 and the library Scipy.stats. A Kruskal–Wallis one-way ANOVA test with Bonferroni correction was used to compare the three setups described above. A *p* < 0.001 was considered a statistically significant difference.

## Study 2: DBS Electrode Implantation

Study 2 assessed the suitability of both EMT systems for the DBS operating room. A DBS surgical theater was created to mimic as faithfully as possible the EM disturbances associated with this environment. During the implantation process, the flextube was used in place of the DBS electrode. Straight implantations were performed for both EMT systems and registered using the Optotrak system.

**Fig. 3:**
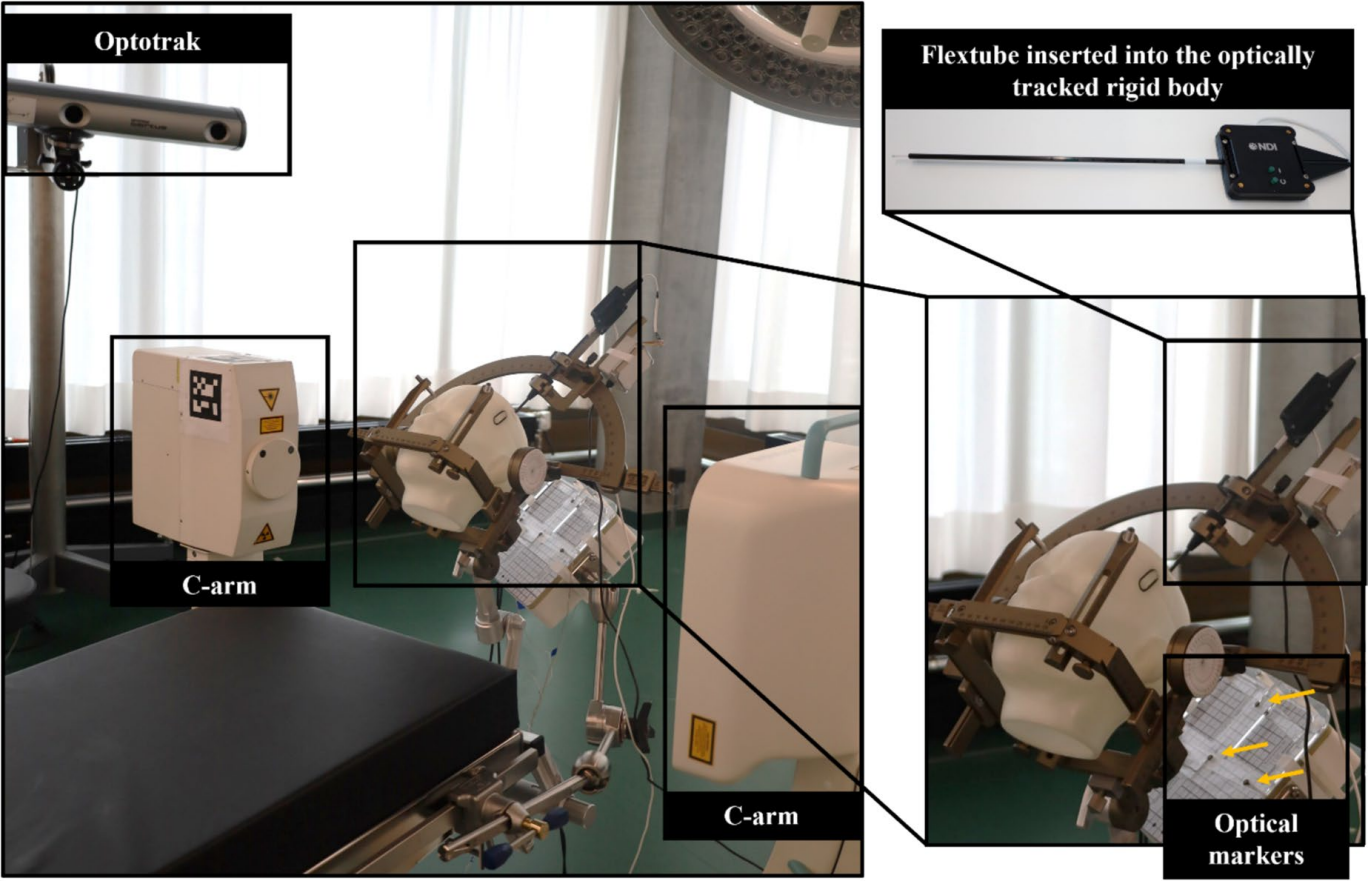
DBS surgical theater: On the left, a picture of the fixation of the EMT system (ManaDBS) and the stereotactic system (G system) to the operating table in the presence of the Optotrak system and the C-arm imager. On the bottom right, the zoom on the stereotactic system with at its bottom left, the ManaDBS FG and the associated optical markers. On the top right, a picture of the rigid body integrating the ManaDBS flextube

**Surgical environment** The simulated operating-theater environment included an operating table along with its requisite accessories: a surgical bed (Maquet Betaclassic, Getinge AB, Gothenburg, Sweden), fixtures for the stereotactic frame comprising a connection bracket (1130.54B0, Getinge AB) and an adjustable base unit (1005.50 A0, Getinge AB), two stereotactic systems with their respective interfaces for the operating table (Leksell Stereotactic System Clamp and Vantage Starburst 3/8”, Elekta AB), and fixations for each EMT system to be attached to the right side rail of the operating table (Figure 3). In contrast to study 1, the FG was placed at the bottom left side of the stereotactic system. A 3D-printed head phantom was positioned within the stereotactic frame. Subsequently, a C-arm (Ziehm Vision FD Vario 3D, Ziehm Imaging GmbH, Nuremberg, Germany) was positioned on either side of the stereotactic arc.

**Reference system** The Optotrak tracking system was again used as the reference system. Four LED markers were fixed to the FG, while a rigid body integrating four embedded LED markers (S-type 4-marker Probe, 8700311, NDI) and the flextube (Figure 3) was used to capture the ground-truth positions and orientations. Subsequently, spatial information was translated into the coordinate system of the FGs.

**Data analysis** Two rectilinear trajectories (one per hemisphere) of 10 cm were defined according to the stereotactic parameters in Table 2. The implantation started from the surface of the phantom head—i.e., the closest of the stereotactic frame—and went down to a depth of 10 cm, further from the stereotactic arc. These two trajectories were reproduced five times for a total of ten trajectories per EMT system and per stereotactic system. Along each trajectory, ten positions were recorded, and for each position 25 measurements were made. In study 2, the systematic error was removed by fitting the two rectilinear trajectories from the optical reference to the two trajectories obtained with the EMT system. An automatic fitting could not be performed with the ICP algorithm as no baseline was available. Therefore, a 3D linear transformation was manually applied. This process eliminated systematic error, leaving only the error associated with trajectory nonlinearity. In image-guided procedures employing EMT or optical tracking, systematic error is typically addressed during the registration step. This step is performed at the beginning of the procedure and involves identifying patient-specific fiducials—such as anatomical landmarks—using both the patient’s imaging and the tracking system. From these fiducials, the spatial transformation between the tracking coordinate space and the patient’s coordinate space is established. To simulate the outcome of this registration step, we manually corrected the systematic error in our study. In this study, the position error was defined as the Euclidian distance between the measured position and the fitted optical reference. Jitter error for each position was defined as previously introduced. Statistical analysis was performed as stated above. The orientation was not investigated due to the missing reference.

**Table 2:** Parameters chosen for the Leksell Frame G and Vantage Frame. These parameters are possible parameters for patients suffering from Parkinson’s disease or essential tremor

	Side	*x*	*y*	*z*	Ring	Arc
	Left	110	98	116	78	112
	Right	88	98	116	78	65

## Results

### Study 1: Tracking Performance in the Presence of a Stereotactic System

**Fig. 4:**
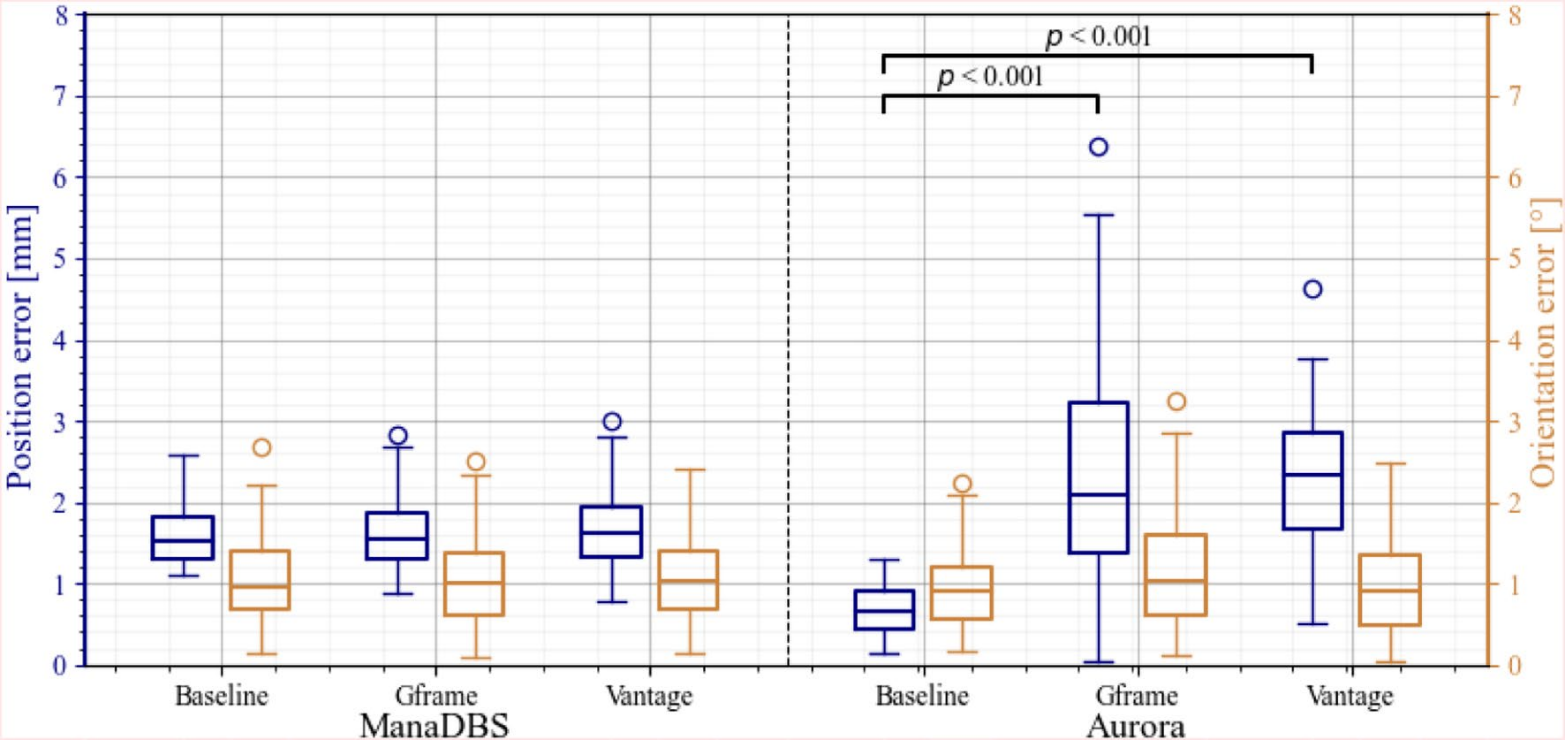
Boxplots of the position (blue boxes on the left) and orientation (yellow boxes on the right) error per setup, calculated from the 100 measurements in the 3D assessment setup. Significant differences are indicated at the top of the boxplots (Color figure online)

On average, for the ManaDBS system, there was no significant difference in the position and orientation errors between the baseline and the stereotactic setups (*p* > 0.2) (Figure 4). The median position errors (Q1–Q3) of the ManaDBS system were about 1.57 mm (1.3–1.9 mm) for all the setups. The median orientation errors (Q1–Q3) were about 1.01° (0.65–1.4°) for all the setups.

For the Aurora system, a significant difference in the position error between the baseline and each stereotactic setup was observed (*p* < 0.001). The median position errors (Q1–Q3) increased from 0.66 mm (0.13–0.89 mm) at baseline to 2.1 mm (1.37–3.24 mm) in the Gframe setup and 2.34 mm (1.67–2.84 mm) in the Vantage setup. There was no significant difference in the orientation errors. However, a slightly high p-value (*p* = 0.07) was observed in the orientation errors between the baseline and the Gframe setup. The median orientation errors (Q1–Q3) were about 0.94° (0.5–1.6°) for all the setups.

There were not any significant differences between the setups for the position jitter (all < 0.8 mm and all < 0.08 mm) and the orientation jitter (all < 0.35° and all < 0.3°) for the ManaDBS and Aurora systems, respectively. The jitter was ten times higher for ManaDBS than Aurora. Additionally, both the position error and the jitter increased at greater distances from the FG, particularly for ManaDBS. These observations can be attributed to the high RMS noise of the sensor used in the ManaDBS system.

**Fig. 5:**
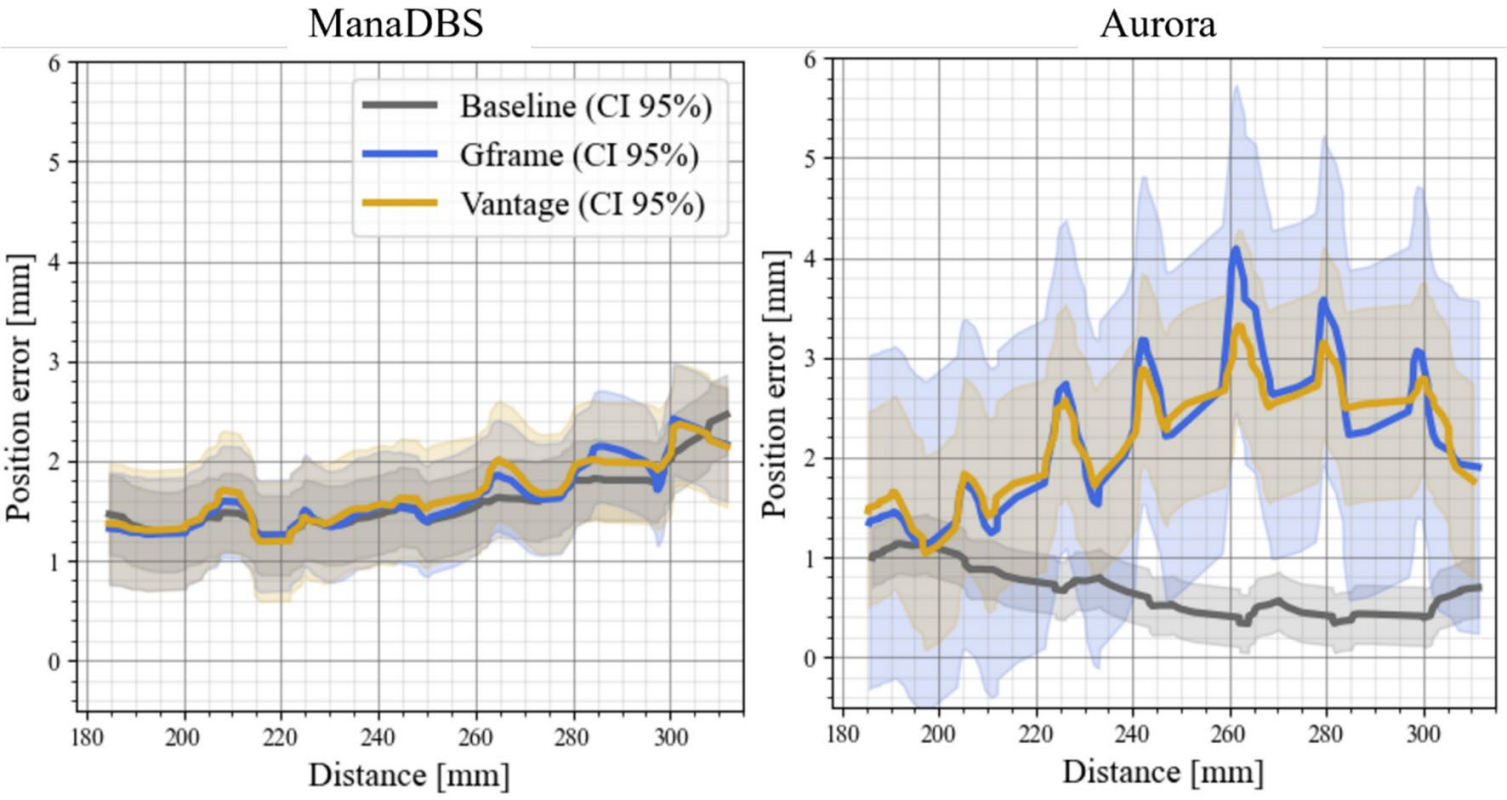
Position error for the ManaDBS system (left) and the Aurora system (right) with 95% confidence intervals as a function of the distance to the FG for the two EMT systems. Data from the three different setups are presented: baseline (gray), Gframe (blue), and Vantage (yellow) (Color figure online)

The position error as a function of the distance of the EMT sensor from the FG for both systems is shown in Figure 5. For ManaDBS, the position error increased by 0.8 mm at greater distances from the FG, but it remained consistent across all the setups. In contrast, the Aurora system exhibited a specific error pattern for the stereotactic setup, with large peak errors at greater distances. These peaks occurred near large metallic parts of the stereotactic systems.

**Fig. 6:**
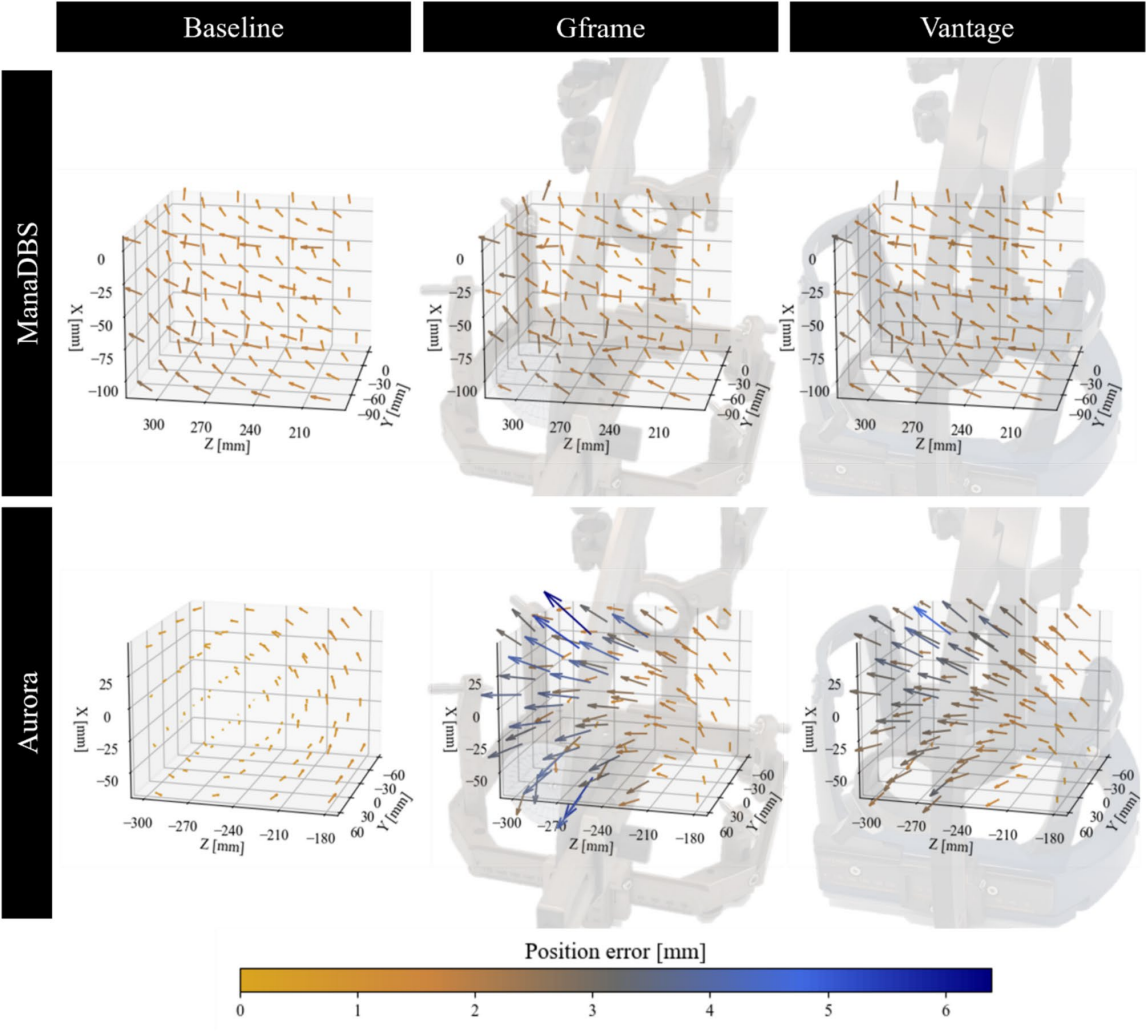
Position error distribution of the ManaDBS (top) and Aurora (bottom) systems for the three setups: baseline (left), Gframe (middle), and Vantage (right). Stereotactic system pictures were transparently added

Figure 6 shows the distribution of position errors in 3D space relative to the placement of the stereotactic systems. The arrow indicates the direction of the error, pointing from the ground-truth position to the measured position. The color of the arrow represents the magnitude of the error. To enhance the visibility of the small error values, the arrow length has been scaled by a factor of 10.

### Study 2: DBS Electrode Implantation

**Fig. 7:**
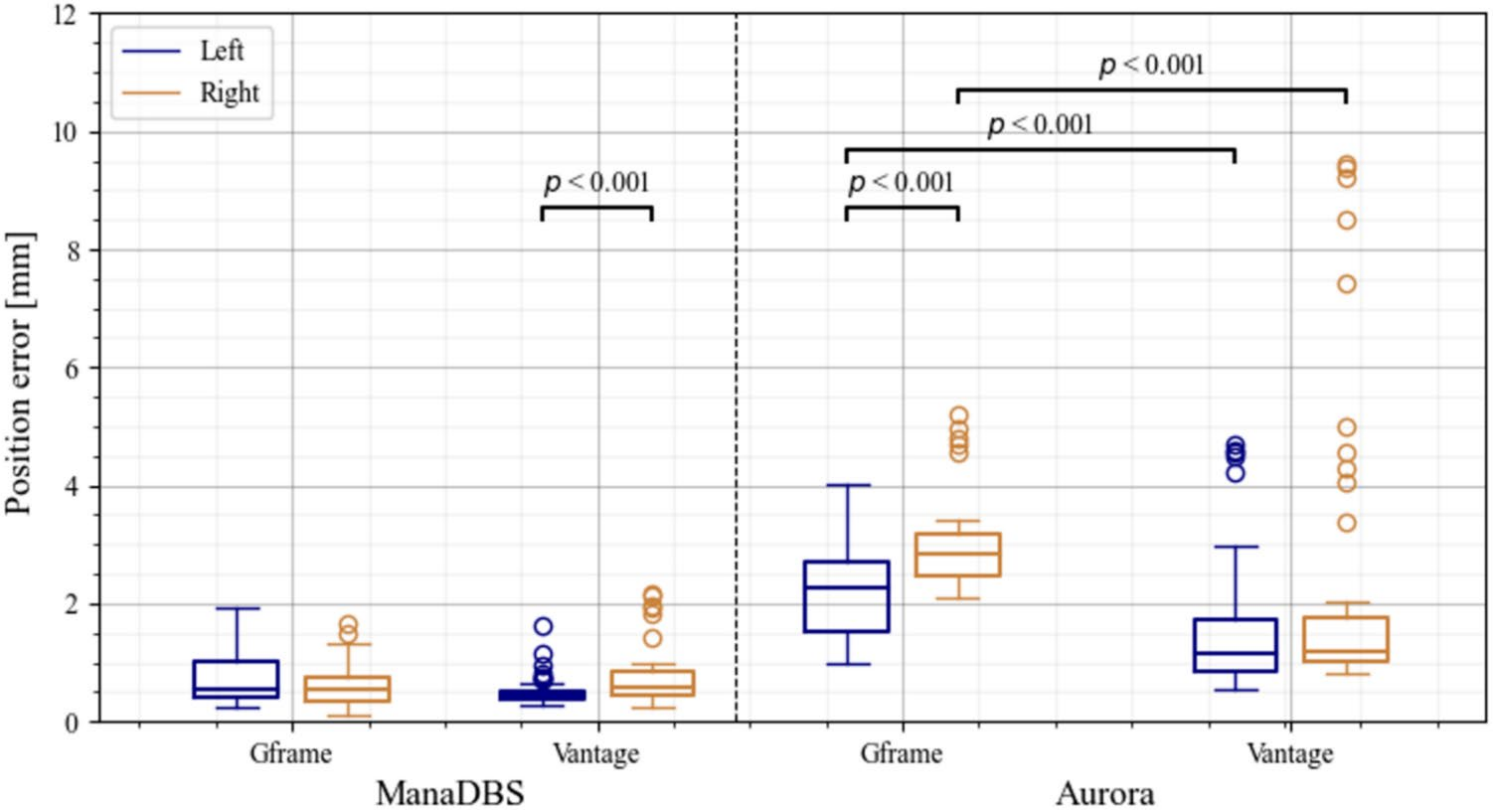
Boxplots of the position errors for the trajectories implanted on the left side (blue boxes on the left) and right side (yellow boxes on the right) per setup. Significant differences are indicated at the top of the boxplots

In Study 2, the position error reflected the nonlinearity of the trajectory, as the systematic errors were removed. Differences were observed based on the implantation side, as shown in the boxplot in Figure 7. For ManaDBS, the median position errors (Q1–Q3) were about 0.55 mm (0.4–0.9 mm) for all the setups. No significant differences between the stereotactic setups were observed regarding the position errors. However, there was a significant difference between the implantation sides for the Vantage setup. Similarly, significant differences between the two implantation sides were observed for the Aurora system. On the left side, the median position errors (Q1–Q3) were 2.3 mm (1.5–2.7 mm) in the Gframe setup and 1.2 mm (0.9–1.7 mm) for the Vantage setup. On the right side, the median position errors (Q1–Q3) increased to 2.8 mm (2.5–3.2 mm) for the Gframe setup and remained about 1.2 mm (1.0–1.7 mm) for the Vantage setup.

There were no significant differences between ManaDBS and Aurora for the position jitter (all < 0.4 mm and < 0.2 mm, respectively) for both the Gframe and Vantage setups.

**Fig. 8:**
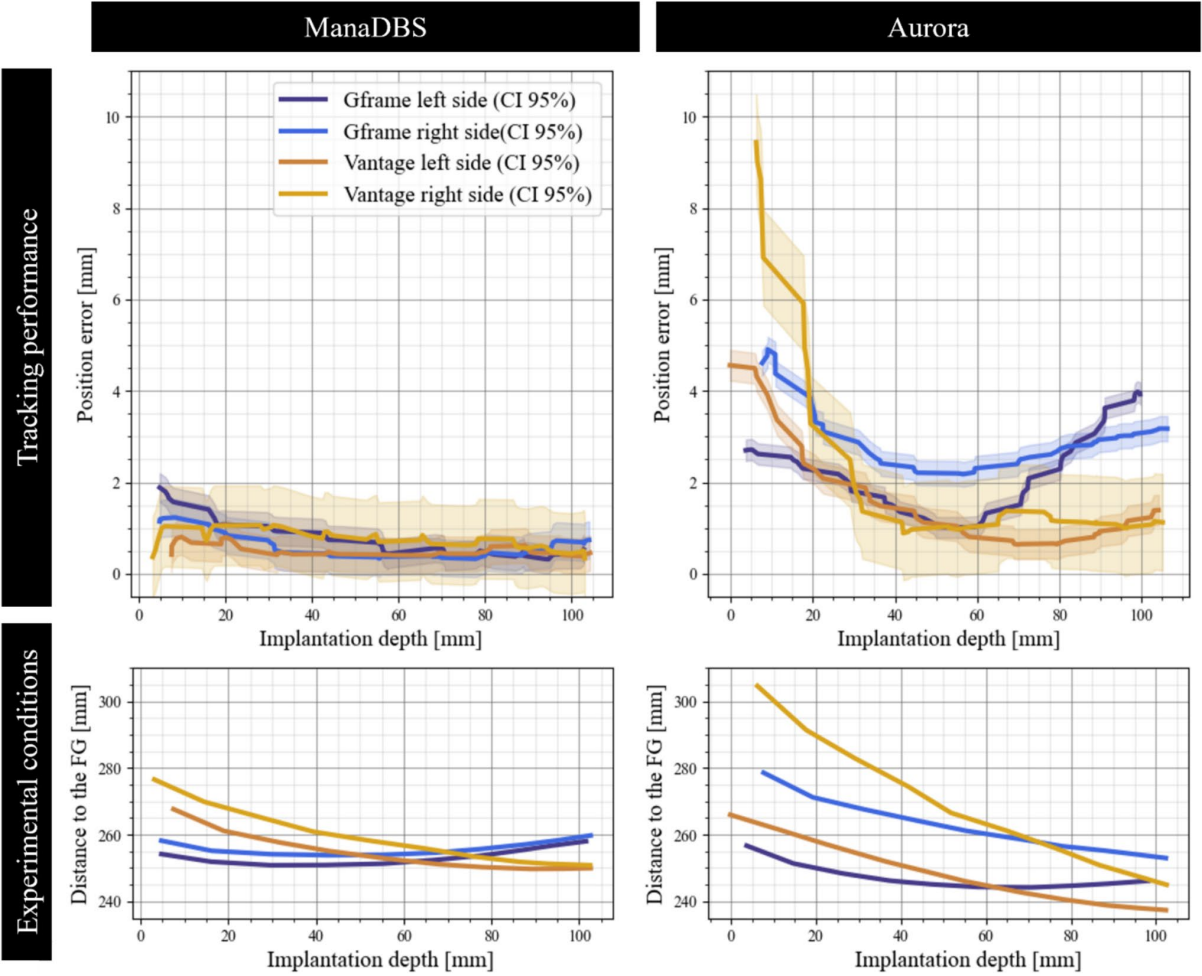
At the top, position errors with 95% confidence intervals as a function of the implantation depth for the ManaDBS system (left) and the Aurora system, (right). Data from the two different stereotactic setups and implantation sides: Gframe left side (dark blue), Gframe right side (light blue), Vantage left side (orange), and Vantage right side (yellow). At the bottom, implantation depth as a function of the distance to the FG

Figure 8 illustrates the position errors relative to the implantation depths of the EMT sensors. The graph also depicts the distance to the FG as a function of implantation depth. The implantations for both EMT systems were performed at similar distances from the FG, ranging from 24 to 30 cm. For ManaDBS, the position errors remained low and consistent across different stereotactic setups. In contrast, the Aurora system exhibited high nonlinear behavior, with errors between 0.6 and 10 mm. The largest errors were observed at the start of the implantations near the stereotactic arc. In the Vantage setup, the position errors decreased with implantation depth. However, in the Gframe setup, the errors initially decreased but subsequently increased.

## Discussion

The DBS surgical theater is a challenging environment that includes many devices such as the operating table, imaging technologies, and stereotactic systems. Providing regular and high-accuracy navigation for the implantation of the DBS electrode has not yet been achieved. When considering factors such as cost, safety, and integration into the surgical environment, electromagnetic navigation emerges as the most suitable technology. However, electromagnetic tracking has only been investigated in frameless settings, which are not the gold standard in DBS surgery [5].

This paper compares the performance of a commercial system and a novel EMT system currently under development. The results indicated that ManaDBS is well-suited for the DBS surgical environment. Study 1 revealed increased localization errors for the Aurora system when a stereotactic system was introduced, whereas ManaDBS maintained consistent localization performance. In study 2, the Aurora system exhibited high nonlinear errors on rectilinear trajectories, with localization errors of up to several millimeters near the stereotactic systems. In contrast, the ManaDBS tracking performance was unaffected, even in a simulated DBS surgical theater. Due to the asymmetry of the experimental setup, differences in performance between the two hemispheres were expected. For the Aurora system, this difference can be partially explained by a greater distance to the FG for the Vantage setup (Figure 8). However, the distance to the FG is not considered the primary cause of this variability; rather, the proximity to the stereotactic system likely contributed to a greater extent. Indeed, the largest errors occurred near the stereotactic arc at the beginning of the implantations. Additionally, in the Gframe setup, increased errors were noted at greater implantation depths. This may be due to the proximity to the stereotactic frame at the end of implantation. The Vantage frame is made from resin, while the Frame G is made from metal, resulting in increased EM perturbations from the Gframe setup. These results confirm the expected performance degradation observed in the Aurora system, which is primarily due to conductive distortions induced by the high-frequency alternating magnetic fields within the stereotactic systems. In contrast, the ManaDBS system generates a quasi-static magnetic field at low frequencies, effectively eliminating these conductive distortions. While the results are promising, further validation is required, as the ManaDBS system has not yet been tested in clinical environments.

This study considers only the effects of non-magnetic metallic tools, such as stereotactic systems, but does not address performance degradation caused by static magnetic distortions from magnetic sources or tools made from ferromagnetic materials. While the offset cancelation step accounts for stray magnetic fields during the activation sequence, it does not mitigate distortions introduced by ferromagnetic objects, which can impact tracking performance. To evaluate the integrity of tracking performance, we tested both EMT systems by positioning various surgical tools (scalpel, retractor, needle holder, and MER electrode…) at distances ranging from 80 to 2 mm from the magnetic sensors (data not presented). Overall, the Aurora system demonstrated better resilience to ferromagnetic tools in position localization (maximal error < 0.3 mm) but showed significant degradation in orientation tracking (ranging from a few degrees at 20 mm to 30 degrees at 2 mm). In contrast, the ManaDBS system was less resilient, with notable impacts on both position and orientation tracking performance (ranging from a few degrees and 0.2 mm at 20 mm to 30 degrees and 1.3 mm at 2 mm). These preliminary results indicate that both systems experienced performance degradation when larger magnetic instruments, such as the retractor and needle holder made of steel, were used. Therefore, a minimum distance of 20 mm from these magnetic tools should be maintained. However, larger tools like retractors and needle holders are typically positioned farther from the DBS electrode in practice. Conversely, the MER electrode, being the closest tool, had no impact on tracking performance in our tests. Currently, commercial systems can detect significant EM perturbations and disable tracking feedback when near distortion sources. A similar approach could be implemented in the ManaDBS system. Further research and development are necessary to better address EM perturbations caused by magnetic sources.

The ManaDBS system is particularly well-suited for DBS procedures, as they involve the gradual insertion of electrodes over several centimeters into the patient’s brain. However, the system’s low update rate should ideally be increased to 2 Hz for better integration and expanded applicability. To address this issue, several improvements can be considered. Using a sensor with lower rms noise and higher frequency would reduce the activation time required for each coil during measurement, enabling an increase in frequency. Alternatively, increasing the magnetic field strength could mitigate the sensor noise, reducing the number of sensor readings needed to maintain tracking performance and thereby increasing the frequency. However, the frequency would still be constrained by the ramp-up time of currents into the coil. Furthermore, the observed spatial position error remained slightly worse than expected for DBS when compared to the performance based on imaging methods (approximately 1–2 mm) [5]. The performance of the ManaDBS system is influenced by the magnetic field strength and the accuracy of current control through the coils that generate the magnetic field. The system’s ability to differentiate between two spatial positions relies on detectable differences in their magnetic fields, which necessitates sufficient sensitivity in the magnetic sensors. In this study, the system’s performance is mainly limited by the rms noise of the sensor, approximately 250 nT, with a resolution of 6.25 nT/LSB. The performance should be enhanced using a sensor with a higher resolution and lower rms noise but also an improved control of the current injected through the FG, and a characterized magnetic field associated with the unique FG magnetic field. The ManaDBS system had not been fully optimized, limiting its actual tracking performance. However, the aim of this study was to compare the compatibility of two EMT systems within a DBS environment.

In addition, the feedback on the position and orientation of the DBS electrode can be coupled with visualization software such as 3D Slicer [21], providing surgeons with visualization of the electrode displacement according to the anatomical brain structures of the patient. Another advantage is the potential mechanical compatibility of the submillimeter magnetic sensor with the DBS electrode. In contrast to the micro-coils used in the commercial system, which typically have a length of 10 mm, rendering them impossible to integrate permanently into the DBS electrode, the magnetic sensor chosen with a robust package of 0.8 × 0.8 × 0.4 mm^3^ should alter the rigidity of the DBS electrode only locally at its tip. The overall length of the DBS electrode should not be affected, allowing the elastic modulus of the DBS electrode to remain close to the brain reducing tissue damage. Providing electrode orientation feedback to the neurosurgeon offers significant advantages, enabling optimized positioning and programming of chronic stimulation. The position and orientation can be transferred to the neurologist to speed up the parametrization process of the directional DBS electrode. While the directional DBS electrode offers substantial benefits by steering the stimulation and expanding the therapeutic stimulation by avoiding adverse effects, it also increases the possible stimulation settings. The ManaDBS system demonstrated an orientation error of 1° across 100 tested positions, which is adequate for DBS application. However, on study 1, only a limited range of orientations was evaluated, further orientations should be tested to cover all possible orientations.

To our knowledge, this paper is the first to quantify the impact of stereotactic systems on the accuracy of an EMT system within a 3D-tracking volume relevant to DBS surgery. Our findings demonstrate that when using Aurora with two different stereotactic systems (Frame G and Vantage, Elekta), there were localization errors of up to 10 mm in position and 1.0° in orientation. In contrast, the ManaDBS system exhibited better compatibility with the DBS environment, achieving an accuracy of approximately 1.7 mm in position and 1.0° in orientation within the clinical working volume. System adaptations are expected to further improve this performance. The results of our studies suggest that navigating DBS electrodes may be feasible using this new generation of EMT system.

## Data Availability

The data are not publicly available. The data that support the findings of this study will be made available by the corresponding author, CV, upon reasonable request.
